# Profil clinico-thérapeutique des états de mal épileptiques dans le service de neurologie de l’Hôpital Befelatanana, Antananarivo: une étude transversale descriptive

**DOI:** 10.11604/pamj.2022.42.118.18726

**Published:** 2022-06-14

**Authors:** Lala Andriamasinavalona Rajaonarison, Nomena Finiavana Rasaholiarison, Jemissair Glorien Lemahafaka, Rahamefy Odilon Randrianasolo, Santatra Razafindrasata, Noël Zodaly, Alain Djacoba Tehindrazanarivelo

**Affiliations:** 1 Service de Neurologie, Hôpital Befelatanana, Antananarivo, Madagascar

**Keywords:** Etat de mal épileptique, Madagascar, STESS, Status epilepticus, Madagascar, STESS

## Abstract

**Introduction:**

l´état de mal épileptique (EME) constitue une urgence diagnostique et thérapeutique. Notre objectif était de décrire le profil clinique et thérapeutique des patients présentant un EME au service de Neurologie de Befelatanana.

**Méthodes:**

c´est une étude rétrospective, descriptive de janvier au juin 2015. Les caractéristiques sociodémographiques, cliniques et thérapeutiques des patients ont été collectées et analysé sur Epi info 7.

**Résultats:**

nous avons retenu 53 patients dont 54,71% étaient épileptiques (n = 29). Les patients de moins de 65 ans prédominaient dans 86,79%. L´âge moyen était de 43,09 ans avec un sex-ratio de 1.30. L´EME convulsif prédominait dans 98,11% (n=52). Il était de type convulsif généralisé dans 66,03%. Le STESS (status epilepticus severity score) inférieur à 3 (77,35%) prédominait. L´électroencéphalogramme (EEG) standard dans les 24h de l´EME était dépourvu d´anomalie épileptique pour tous les patients. Une rupture de traitement antiépileptique (9,43%) et une privation de sommeil (18,86%) ont été rapportées comme facteur déclenchant de l´EME. Une absence de crise épileptique dans les 72h de la mise sous notre protocole était notée dans 84,90% des cas. Nous n´avons pas trouvé d´association significative entre le statut épileptique ou non avec le STESS (p = 0,302), le protocole de traitement (p = 0,532) et la rémission des crises dans les 72 heures (p = 0,211).

**Conclusion:**

l´EME touche une population jeune et épileptique. Notre protocole a permis une rémission des crises dans les 72h dans la plupart des cas. Une étude sur la validation de ce protocole d´EME est nécessaire.

## Abstract (en)

**Introduction:**

Status Epilepticus (SE) is a diagnostic and therapeutic emergency. The purpose of this study was to establish the frequency, the clinical and therapeutic features of patients with SE at the Department of Neurology of Befelatanana.

**Methods:**

we conducted a retrospective, descriptive study from January to June 2015. The sociodemographic and clinical features of patients were collected and analyzed on Epi info 7.

**Results:**

the study involved 53 patients, 54.71% of whom were epileptic (n=29). There was a predominance of patients under 65 years of age (86.79%). The average age of patients was 43.09 years with a sex-ratio of 1.30. Convulsive SE prevailed in 98.11% of cases (n=52). Generalized convulsive SE occurred in 66.03% of cases. STESS below 3 (77.35%) predominated. There were no epileptic abnormalities on standard EEG within 24 hours in all patients with SE. Non-adherence to antiepileptic therapy (9.43%) and sleep deprivation (18.86%) were reported as a trigger factor of SE. No seizure was reported for up to 72 hours after initiation of treatment in 84,90% of cases. We found no significant association between epileptic or non-epileptic status and STESS (p = 0.302), treatment protocol (p = 0.532), and 72-hour remission of seizures (p = 0.211).

**Conclusion:**

SE affects young and epileptic people. Our treatment protocol allowed for crisis remission within 72h in most cases. A validation study about this therapeutic protocol is required.

## Introduction

Les états de mal épileptiques (EME) représentent l´expression maximale de l´épilepsie [1]. Ils constituent la première manifestation de l´épilepsie chez environs 50% des patients [2]. L´incidence annuelle est évaluée de 10 à 41 pour 100 000 habitants [3]. L´incidence est plus élevée chez les patients épileptiques (50%), les jeunes enfants et les sujets âgés [4]. Si le diagnostic d´état de mal épileptique peut sembler simple à priori, cette entité électro-clinique regroupe un ensemble hétérogène de présentation clinique pouvant conduire à méconnaitre le diagnostic, ou, au contraire à le porter à tort [5]. Les EME sont des urgences médicales majeures associés à un taux de mortalité qui varie selon plusieurs paramètres. La mortalité liée à l´état de mal épileptique de la phase aiguë au 30^e^ jour varie de 3 à 39% selon les études tandis que la mortalité à long terme varie de 17 à 80% [6]. L´insuffisance de donnée épidémiologique pour Madagascar nous a incités à réaliser cette étude. Notre étude visait à décrire le profil clinique et thérapeutique de cette l´EME dans le service de Neurologie du Centre Hospitalo-Universitaire Joseph Raseta Befelatanana.

## Méthodes

**Conception et contexte de l´étude**: il s´agit d´une étude rétrospective descriptive et transversale visant à décrire le profil clinico-thérapeutique des patients présentant un état de mal épileptique auprès du service de Neurologie du Centre Hospitalo-Universitaire Joseph Raseta Befelatanana (CHU JRB). Elle se déroulait du 01 janvier 2015 jusqu´au 30 juin 2015 (6 mois).

**Population d´étude**: nous avons inclus tout dossier de patient ayant comme diagnostic de sortie auprès du service de Neurologie du CHU JRB un état de mal épileptique chez un patient de plus de 18 ans avec ou sans scanner cérébral. Nous avons exclu tout dossier de patient incomplet. Nous avons recherché une association entre le STESS, le protocole de traitement et la rémission des crises dans les 72 heures selon le statut épileptique ou non de nos patients. Les patients épileptiques étaient les patients ayant un antécédent de crise épileptique de plus de 24 heures avant son état de mal épileptique et les patients non épileptiques étaient ceux qui étaient hospitalisés pour une première crise épileptique en état de mal.

**Collecte des données**: le recueil des données a été réalisé à l'aide d'une fiche d'exploitation, visant à préciser les aspects sociodémographiques, cliniques et thérapeutique des patients avec un état de mal épileptique. Nous avons relevé les paramètres à étudier dans les dossiers des patients. Ils étaient présentés en valeur absolue et en pourcentage. La fréquence absolue, la fréquence relative (par rapport au genre et à l´âge, le statut épileptique ou non épileptique), les variables socio-démographiques (l´âge et le genre), les variables cliniques (le statut épileptique; le type d´état de mal épileptique (convulsif ou non convulsif); le STESS (Status Epilepticus Severity Score: STESS < 3 ou STESS ≥ 3), le variable paraclinique: EEG (électro-encéphalographie), le traitement des patients. Notre thérapeutique était basée sur une perfusion continue de Diazépam en 24 heures à la posologie de 0,3 à 0,5 mg/kg/j après un bolus de Diazépam 10 mg en intraveineuse directe lente (IVDL). La perfusion était maintenue jusqu´à 72 heures sans crises. Nous avons associé d´emblée un antiépileptique (la Carbamazépine par voie orale à la posologie progressive d´administration jusqu´à dose thérapeutique de 10 à 15 mg/kg/j) à la perfusion. En cas de persistance des crises nous avons eu recours à un bolus de Phénobarbital puis le transfert en service de réanimation médicale.

**Méthode statistique**: le logiciel Excel 2007 et l´Epi info 7 ont été utilisé pour traiter nos données statistiques. Les résultats sont représentés en valeur absolue, en pourcentage et en moyenne. Pour l´étude de corrélation, une valeur de p < 0,05 était considérée comme statistiquement significative.

**Considération éthique**: les secrets professionnels et médicaux ont été respectés ainsi que l´anonymat des patients en utilisant des codes.

## Résultats

Nous avons colligé 53 cas ayant comme diagnostic de sortie un état de mal épileptique sur 313 patients pendant les 6 mois de l´étude (16,93%). Aucun patient n´a été exclu. La majorité de la population d´étude faisait partie des moins de 65 ans (86,79%). L´âge moyen était de 43,09 ans avec un extrême de 19 ans et 81 ans. Le genre masculin prédominait (n= 30) avec un sex-ratio de 1,30 (Tableau 1). Vingt-neuf patients (54,71%) étaient épileptiques dont 12 patients n´ont pas été sous antiépileptique au long cours (Tableau 1). Le diagnostic d´EME a été posé selon les critères cliniques pour 52 patients (98,11%) suite à un EME de type convulsif. Il était de type convulsif généralisé d´emblée (n=35), partiel (n=8), partiel secondairement généralisé (n=5), tonique (n=3) et clonique (n=1). Dans le groupe des patients épileptiques (n=29), l´EME survenait surtout chez ceux qui ont une épilepsie de type généralisé dans 68,96% (n = 20). L´EME était surtout de type convulsif généralisé dans 75,86% (n=22). Tous les patients non épileptiques (n = 24) présentaient un EME de type convulsif avec une prédominance de la forme convulsive généralisée dans 66.66 % (n = 16). Quarante et un patients ont eu un STESS < 3 (77,35%).

**Tableau 1:** fréquence absolue et fréquence relative par rapport au sexe, âge et épilepsie

	N	Pourcentage(%)
**Fréquence absolue**	53/313	16,93
**Fréquence relative**		
**Age**		
< 65 ans	**30/160**	**18,75**
≥ 65 ans	23/153	15,03
**Genre**		
Masculin	**46**	**86,79**
Féminin	7	13,20
Epilepsie*		
Oui	**29**	**54,71**
Non	24	45,28

* épileptique ou pas

L´EEG a confirmé un EME non convulsif dans notre étude (1,88%). Tous nos patients ont eu un EEG standard dans les 24h de la prise en charge de leur EME. Aucune anomalie paroxystique de la série épileptique n´a été notée. Une rupture de traitement antiépileptique de plus de 48h (9,43%) et une privation de sommeil la veille de l´EME (18,86%) ont été rapportées comme facteur déclenchant de l´EME dans notre étude. Tous nos patients ont été mis sous notre protocole d´EME. L´évolution a été marquée par une absence de crise épileptique à plus de 72h (84,90%), une persistance des crises épileptiques (3,77%) et un transfert en service de réanimation (1,88%). Nous n'avons pas trouvé d´association significative entre le statut épileptique ou non avec le STESS (p = 0,302), le protocole de traitement (p = 0,532) et la rémission des crises dans les 72 heures (p = 0,211) (Tableau 2).

**Tableau 2:** mesure d´association entre statut épileptique ou non et variables de l´étude

Variables	Total (N = 53)	Epileptique oui (n = 29)	Epileptique non (n = 24)	p
Type convulsifï	52 (98,11%)	28 (96,55%)	24 (100%)	0,358
Forme généralisée	38 (71,69%)	22 (75,86%)	16 (66,66%)	0,459
Diagnostic clinique	52(98,11%)	28(96,55%)	24(100%)	0,358
Privation de sommeil	10(18,86%)	6(20,68%)	4(16,66%)	0,709
STESS ≥ 3	12 (22,64%)	5 (17,24%)	7 (29,16%)	0,302
Bénéfice d´un EEG	53 (100%)	29 (100%)	24 (100%)	
Protocole EME	47 (88,67%)	25 (86,31%)	22 (91,66%)	0,532
**Absence de crise ≥ 72h**	45(84,90%)	23(79,31%)	22(91,66%)	0,201

STESS: Status Epilepticus Severity Scale

## Discussion

Dans notre étude, plus de la moitié était des épileptiques, avec une population relativement jeune, qui présentait surtout un EME généralisé convulsif, déclenché par rupture de traitement antiépileptique et une privation de sommeil dans la majorité des cas. La plupart avait un STESS inférieur à 3. Ils n´avaient pas d´anomalie à l´EEG standard dans les 24h de l´EME. Après 72h de notre protocole de traitement une absence de crise épileptique était noté. Nous n'avons pas trouvé d´association significative entre le fait d´être épileptique ou non avec le STESS, le protocole de traitement et la réponse au traitement. Même si notre étude était parmi les premières études sur l´état de mal épileptique et son traitement à Madagascar, elle a ses limites, elle est monocentrique, avec un petit nombre de population dont le traitement a été fait par un protocole que seul notre service utilisait lors de l´étude. Nous ne pouvons généraliser pour avoir une prévalence ou incidence de l´état de mal épileptique à Madagascar à partir de notre étude et l´application de notre protocole de traitement d´EME nécessite encore un grand nombre de population et une comparaison d´efficacité avec un autre protocole.

Nous avons rapporté 53 cas d´EME durant les 6 mois de l´étude. L´âge moyen de nos patients était de 43,09 ans avec une prédominance du genre masculin. Une différence du nombre de population recruté a été notée selon la durée de l´étude, le site choisi et les critères d´inclusion. Raveloson *et al*. rapportait 66 cas d´EME sur 6 mois auprès d´un service d´Accueil, Triage et Urgence sur une population adulte [7]. Quant à Doumbia-Quattara *et al*. ils ont recruté sur une période de 62 mois 32 dossiers de patients présentant un EME incluant à la fois une population pédiatrique [8]. Raveloson N *et al*. a retrouvé un âge moyen de 44,54 ans [7]. La prédominance masculine est rapportée aussi par Ong C-T *et al*. avec un sex-ratio de 1.57 [9] de même que Raveloson *et al*. avec une population majoritairement masculin (62%) [7]. Les caractéristiques démographiques de notre population d´étude peuvent s´expliquer par la jeunesse de la population Malagasy selon l´enquête sur le ménage à Madagascar [10]. L´EME convulsif prédominait dans 98,11% (n=52). Il était de type convulsif généralisé dans 66,03%. Ce qui a facilité de porter le diagnostic clinique de l´EME pour nos patients (n=52). Plusieurs études s´accordent à dire que l´EME de type convulsif généralisé prédominait. Ils étaient de l´ordre de 83,3% dans l´étude de Ramamonjisoa *et al*. [11] et de 70% pour Doumbia-Quattara M *et al*. [8]. Cette hausse de la forme convulsive généralisée pourrait s´expliquer par la facilité à poser le diagnostic selon les critères cliniques: devant la manifestation clinique, la durée des crises, l´antécédent d´épilepsie ou l´existence de cas similaire antérieur. Ceci basée sur la nouvelle définition et classification par ILAE en 2015 par Trinka *et al*. [12].

Le STESS inférieur à 3 (77,35%) prédominait dans notre étude. Plusieurs facteurs influençant le pronostic ont été décrits dans la littérature dont le type de crises au moment de l´état de mal selon Rossetti *et al*. (2006) [13]. Dans leur étude, un score STESS < 3 a une valeur prédictive négative du décès à 97 % et qu´un score de STESS < 3 est fortement corrélé à la survie du patient [13]. Un EME non convulsif a été posé après la réalisation de l´EEG. L´EEG permet devant un EME d´aider à poser le diagnostic positif surtout en cas d´EME non convulsif ou en cas de crises infra-cliniques et d´éliminer les autres diagnostics différentiels [14]. La présence d´activité rythmique est une des caractéristiques de crise à l´EEG. Cisse A *et al*. avaient noté la présence de tracé pathologique (focal ou généralisé) dans 62,23% de cas de ses patients [15]. L´EEG permet aussi de faire un suivi thérapeutique du patient. Dans notre étude, l´EEG standard dans les 24h de l´EME était dépourvue d´anomalie épileptique pour tous nos patients. Elle permet de rechercher un EME réfractaire ou larvé ou d´autre protocole thérapeutique est à mettre en place [14]. Nous avons rapporté deux facteurs déclenchant l´EME de nos patients à savoir la rupture de traitement antiépileptique (9,43%) et la privation de sommeil (18,86%). Randrianantoandro *et al*. ont rapporté à part la rupture de traitement (18,1%) et la privation de sommeil (13,8%) d´autres facteurs comme les sevrages/intoxication éthylique (18,1%), métaboliques (11,6%) ou des encéphalites (2,9%) [16]. La difficulté d´accès à une imagerie cérébrale nous a limité à rechercher les lésions cérébrales épileptogènes qui représente 24% des étiologies d´EME dans les pays développés. Tandis que les infections cérébro-méningés occupent une place importante dans l´étiologie d´EME dans les pays d´Afrique subsaharienne [17].

La prise en charge médicale de nos patients était basée sur une perfusion de Diazépam (0,5 à 1mg/kg/24h) après un bolus d´une ampoule de 10 mg. La perfusion est maintenue jusqu´à 72 heures sans crises. On y associe d´emblée la Carbamazépine par voies orale (à travers une sonde nasogastrique) à la posologie progressive d´administration de 5 mg/kg/j jusqu´à 10 à 15mg/kg/j. Les patients sont hospitalisés en soins d´urgence en Neurologie et bénéficient d´une surveillance EEG journalière. Cissé A *et al*. avaient rapporté un schéma thérapeutique assez identique à la nôtre. Ils avaient eu recours à la benzodiazépine comme traitement de première intention (93,33%). Face à la persistance des crises dans 90% des cas, l´adjonction d´un traitement antiépileptique (intra-veineuse ou per os par une sonde nasogastrique) avec la perfusion d´une benzodiazépine répartie en trois prises dans la journée [15]. L´accessibilité limitée de nos patients aux antiépileptiques qui sont à leur charge nous oblige à élaborer des protocoles simples, efficaces et qui coutent moins chères. L´absence de crise épileptique dans les 72h (84,90%) de la mise sous notre protocole n´était pas associée significativement au statut épileptique ou non de nos patients (p=0,211). L´efficacité thérapeutique ne dépend pas du statut épileptique ou non du patient comme rapporte aussi l´étude de Alahmari ZS *et al*. (p=0,811) [18]. C´est une urgence médicale absolue. L´instauration rapide du traitement selon le stade de l´EME en est un des facteurs d´efficacité thérapeutique [19]. A la fin de notre étude nous suggérons l´utilisation du STESS score dans la classification de la sévérité de l´état de mal épileptique. L´efficacité de notre protocole de traitement et sa validation a encore besoin d´une étude sur une grande échelle pour qu´il devienne une recommandation nationale.

## Conclusion

L´EME dans notre étude affecte une population jeune à prédominance masculine. Il est de type convulsif généralisé dans la majorité des cas. La rupture de traitement antiépileptique et la privation de sommeil fait partie des facteurs favorisants la survenue de l´EME. La plupart des patients avaient une rémission de leur EME après notre protocole de traitement. Face à la difficulté de la mise en pratique des recommandations internationales sur l´EME à Madagascar, la validation à travers des études multicentriques de notre protocole pourrait aider à la prise en charge des EME dans les zones enclavées de l´île.

### Etat des connaissances sur le sujet

- La plupart des données concernant les états de mal épileptiques viennent des pays non africains;
- Le Clonazepam est utilisé en première intention dans la prise en charge des états de mal épileptiques (EME);
- Les états de mal épileptiques sont surtout pris en charge en réanimation.

### Contribution de notre étude à la connaissance

- Des données à propos des états de mal épileptiques, utilisant le score de STESS dans l´évaluation de la gravité de ce dernier dans un pays africain comme Madagascar;
- L´utilisation de Diazepam en première intention dans le traitement des états de mal épileptiques;
- La prise en charge d´état de mal épileptique dans un service de neurologie.
